# Complete Genome Sequence of *Bdellovibrio* sp. Strain KM01, a Predatory Bacterium Isolated from Soil

**DOI:** 10.1128/MRA.00838-20

**Published:** 2020-10-01

**Authors:** Sidney C. Davis, Karla J. Martinez, Laura E. Williams

**Affiliations:** a Department of Biology, Providence College, Providence, Rhode Island, USA; Indiana University, Bloomington

## Abstract

We report the complete genome sequence of the predatory bacterium *Bdellovibrio* sp. strain KM01, isolated from soil collected near a pond. The genome is 3,961,288 bp long with 45.5% GC content. Comparative genomics among *Bdellovibrio* strains will help us understand how genotypic differences affect differences in predatory phenotypes.

## ANNOUNCEMENT

Because *Bdellovibrio* species can kill Gram-negative bacteria, they may be useful as clinical therapies; however, more *Bdellovibrio* genome sequences are needed to understand how genotype affects differences in predatory phenotypes. We report the genome of *Bdellovibrio* sp. strain KM01, isolated following the enrichment protocol described in reference 1 from soil collected near a pond at a Rhode Island state park.

To obtain genomic DNA, we cocultured KM01 in HM buffer (1) with 1.5 ml overnight culture of *Raoultella* sp. strain 0037. After 48 h at 28°C and 200 rpm, we passed the coculture through a 0.45-μm filter and then extracted genomic DNA from the filtrate containing *Bdellovibrio* sp. strain KM01 using the Wizard genomic DNA purification kit (Promega). The University of Maryland Institute for Genome Sciences sheared genomic DNA using a g-TUBE at 3,400 rpm and then size selected it on a Blue Pippin instrument (Sage Scientific) with an 11,000-bp cutoff. The library was prepared using a SMRTbell template prep kit v1.0 with no modifications and sequenced on a PacBio RS II instrument with P6-C4 chemistry. The University of Rhode Island Genomics and Sequencing Center sheared genomic DNA using a Covaris S-220 focused ultrasonicator. The library was prepared using a PrepX DNA library kit (TaKaRa Bio), visualized on a high-sensitivity BioAnalyzer chip (Agilent), and quantified using the KAPA Illumina quantification kit (Roche). The library was sequenced on an Illumina MiSeq instrument to generate 2 × 250-bp paired-end reads.

For *de novo* assembly of long reads, we launched an Amazon EC2 instance of SMRT Portal v2.3.0 and used Hierarchical Genome Assembly Process v3 (HGAP3) (2) with default target coverage (25×) and 4.0 Mb estimated genome size. From one single-molecule real-time (SMRT) cell, we obtained 99,389 subreads with an *N*_50_ value of 12,098 bp, which assembled into a 3,978,353-bp contig with 158× mean coverage. To circularize the contig, we used BLAST (3) to locate the overlap between contig ends and EMBOSS extractseq (4) to trim the overlap, yielding a 3,960,707-bp closed contig. Using extractseq, we adjusted the first position to align it with the predicted *dnaA* start codon.

To polish the closed contig, we processed 2,028,031 MiSeq read pairs with SolexaQA v3.1.4 (5) using DynamicTrim to remove bases with a quality score of <13 and then using LengthSort to discard reads of <65 bp. This yielded 1,925,830 read pairs. Using the Burrows-Wheeler Aligner MEM algorithm (BWA-MEM) v0.7.13 (6), we mapped 98% of the reads (3,775,952 of 3,851,660) to the closed contig. We sorted and indexed the alignment with SAMtools v1.3 (7) and then confirmed 99.98% of the closed contig using Pilon v1.22 (8). Pilon identified and corrected 584 small indels and 1 single-nucleotide polymorphism (SNP), and it flagged five regions as local continuity breaks, two of which it fixed. This yielded a 3,961,296-bp draft sequence.

To evaluate continuity breaks and assess changes made by Pilon, we used Canu v2.0 (9) to correct PacBio subreads and then used minimap2 v2.1 (10) to align corrected reads to the draft sequence. We sorted and indexed the alignment with SAMtools and then visualized it with Tablet v1.19.09.03 (11). Within the regions previously flagged by Pilon as local continuity breaks, we examined the alignment for variants and corrected six homopolymer tracts, generating the final 3,961,288-bp genome with 45.5% GC content and 160× mean MiSeq read coverage.

We annotated the genome with Rapid Annotations using Subsystems Technology (RAST) (12) and NCBI’s Prokaryotic Genome Annotation Pipeline (PGAP) (13). PGAP identified 3,752 protein-coding genes, 44 RNA-coding genes, and 17 pseudogenes.

### Data availability.

The *Bdellovibrio* sp. strain KM01 genome sequence was deposited in GenBank under accession number CP058348. The MiSeq and PacBio reads were deposited in the SRA under accession numbers SRR12207263 and SRR12207262, respectively.
